# Healing beyond medicine: Effect of nurse-led body-mind-spirit interventions on well-being in women undergoing infertility treatment

**DOI:** 10.6026/973206300213342

**Published:** 2025-09-30

**Authors:** Amudha Chinnappan, Sumathi Chandran, Shankar Shanmugam Rajendran, Vanitha Narayanasamy Naidu, Azhgar Jan Thoulath Khan, Atchaya Rajagopal, Jessy Raja

**Affiliations:** 1 Department of Obstetrics and Gynecological Nursing, College of Nursing, Madras Medical College, The Tamil Nadu Dr. MGR Medical University, Chennai, Tamil Nadu, India; 2 Department of Obstetrics and Gynecology, Institute of Obstetrics and Gynaecology, Government Hospital for Women and Children, Madras Medical College, The Tamil Nadu Dr. MGR Medical University, Chennai, Tamil Nadu, India; 3 Department of Child Health Nursing, College of Nursing, Madras Medical College, The Tamil Nadu Dr. MGR Medical University, Chennai, Tamil Nadu, India; 4 Department of Community Health Nursing, College of Nursing, Madras Medical College, The Tamil Nadu Dr. MGR Medical University, Chennai, Tamil Nadu, India

**Keywords:** Nurse-led body-mind-spiritual intervention, women undergoing infertility treatment, psychological well-being, hope

## Abstract

The emotional distress and diminished hope experienced by women undergoing infertility treatment is a significant challenge. Employing a true experimental design, the study involved 100 participants selected through simple random sampling. The experimental group participated in a structured four-week BMS program that included physical exercises, guided meditation, reflective writing, prayer and spiritual self-exploration. Follow the intervention, a significant improvement (p < 0.05) in both psychological well-being and levels of hope. Thus, we show the importance of integrating holistic nursing approaches to support emotional resilience in women facing infertility challenges.

## Background:

Infertility is a multifactorial condition that affects not only a woman's physical ability to conceive but also her psychological and spiritual health [1]. In India, the rising trend is attributed to changing lifestyles, delayed marriages and exposure to environmental pollutants, with approximately 10-15% of couples experiencing infertility [2]. Women undergoing infertility treatment often suffer silently with psychological distress such as anxiety, depression, low self-worth and loss of hope, which are rarely addressed in routine care [3]. Conventional medical interventions primarily target reproductive outcomes while neglecting the emotional and spiritual needs of the patient [4]. Nurse-led body-mind-spiritual interventions offer holistic care by integrating physical, emotional and spiritual support. Activities such as meditation, journaling, altruistic acts and prayer have been shown to enhance psychological well-being and promote optimism [5]. This study was inspired by clinical observations where many infertile women appeared emotionally exhausted and in need of structured support. Therefore, it is of interest to explore the effectiveness of nurse-led holistic interventions in reducing emotional distress and enhancing hope during infertility treatment.

## Methodology:

A true experimental research design was employed for this study, which was conducted at the Infertility Clinic at IOG, Egmore, Chennai, following ethical clearance obtained on November 19, 2024. A simple random sampling method was used to select 100 women undergoing infertility treatment, who were then evenly divided into two groups: 50 participants were assigned to the experimental group, and 50 to the control group. The inclusion criteria consisted of women undergoing treatment, who were available during data collection, and who were willing to provide informed consent. Exclusion criteria included women who were critically ill, uncooperative, or unwilling to participate. The experimental group received a structured one-month Body-Mind-Spiritual (BMS) intervention, which incorporated physical practices (breathing exercises, diet awareness, and massage), mental practices (journaling and meditation), and spiritual practices (prayer, self-reflection, and altruism). The control group received routine infertility care. Psychological well-being and hope were assessed using the Ryff & Keyes Psychological Well-being Scale (18 items, 7-point Likert scale; score range: 18-126) and the Herth Hope Index (12 items, 4-point scale; score range: 12-48). Subject experts validated both tools, and their reliability was confirmed (Cronbach's alpha: 0.87 for well-being, 0.84 for hope). Data were analyzed using SPSS software, with a significance level of p ≤ 0.05 to determine statistical relevance.

## Results:

Most participants were aged 26-30 years (40%), had studied up to higher secondary level (38%) and had experienced infertility for 1 to 5 years. After receiving the nurse-led body-mind-spiritual (BMS) intervention, the experimental group showed a marked improvement in psychological well-being, with mean scores rising from 15.8 to 31.40 (mean difference = 15.60, p = 0.001). The control group showed only slight improvement (mean difference = 7.13, p = 0.06) (Table 1 - see PDF). Similarly, hope levels significantly increased in the experimental group (mean difference = 8.97, p = 0.001), while the control group showed minimal change (p = 0.07) (Table 2 - see PDF). A strong positive correlation was found between psychological well-being and hope (r = 0.58, p = 0.001) in the intervention group. Although better outcomes were seen in younger, more educated women with shorter infertility durations, these differences were not statistically significant in the control group.

## Discussion:

This study found that nurse-led body-mind-spiritual care significantly boosted psychological well-being and hope in women facing infertility. Similar results were noted by Yanik and Budak (2024) [6], who showed that positive therapy improved mental health and hope. The strong link between well-being and hope (r = 0.58, p = 0.001) also supports Szigeti *et al.* (2024) [7], who stressed the role of emotional strength in reproductive health. Participants' emotional struggles, including stress and feelings of inadequacy, are consistent with observations by Paulus *et al.* (2021) [8], who highlighted the need for holistic nursing care that addresses both emotional and spiritual dimensions. The effectiveness of the intervention also echoes Weis (2011) [9] support for guided hope practices in clinical settings. Incorporating journaling, meditation, prayer and acts of kindness appeared to help women cope better, a finding similar to those reported by Keng (2011) [10], who found improved self-concept with spiritual interventions. While the study focused on short-term outcomes, it highlights the importance of integrating holistic, nurse-led programs into infertility care. Future research should explore long-term effects and the influence of family and partner support.

## Conclusion:

A nurse-led body-mind-spirit approach led to a marked enhancement in both psychological well-being and hope among women receiving infertility care. This holistic approach helped them manage emotional stress and develop a more positive mindset. Thus, we show the need to include nurse-led emotional and spiritual care as part of routine infertility treatment to support overall recovery.
